# A randomized comparative study of patients undergoing myocardial revascularization with or without cardiopulmonary bypass surgery: The MASS III Trial

**DOI:** 10.1186/1745-6215-9-52

**Published:** 2008-08-28

**Authors:** Whady Hueb, Neuza HM Lopes, Bernard J Gersh, Cláudio C Castro, Felipe S Paulitsch, Sergio A Oliveira, Luis A Dallan, Alexandre C Hueb, Noedir A Stolf, José AF Ramires

**Affiliations:** 1 Department Clinical Heart Institute of the University of Sao Paulo, Sao Paulo, Brazil; 2 Department Cardiovascular Diseases Mayo Clinic, Rochester, MN, USA

## Abstract

The MASS III Trial is a large project from a single institution, The Heart Institute of the University of Sao Paulo, Brazil (InCor), enrolling patients with coronary artery disease and preserved ventricular function. The aim of the MASS III Trial is to compare medical effectiveness, cerebral injury, quality of life, and the cost-effectiveness of coronary surgery with and without of cardiopulmonary bypass in patients with multivessel coronary disease referred for both strategies. The primary endpoint should be a composite of cardiovascular mortality, cerebrovascular accident, nonfatal myocardial infarction, and refractory angina requiring revascularization. The secondary end points in this trial include noncardiac mortality, presence and severity of angina, quality of life based on the SF-36 Questionnaire, and cost-effectiveness at discharge and at 5-year follow-up. In this scenario, we will analyze the cost of the initial procedure, hospital length of stay, resource utilization, repeat hospitalization, and repeat revascularization events during the follow-up. Exercise capacity will be assessed at 6-months, 12-months, and the end of follow-up. A neurocognitive evaluation will be assessed in a subset of subjects using the Brain Resource Center computerized neurocognitive battery. Furthermore, magnetic resonance imaging will be made to detect any cerebral injury before and after procedures in patients who undergo coronary artery surgery with and without cardiopulmonary bypass.

Clinical Trial registration information

ISRCTN59539154 Off-pump vs. on-pump surgery in patients with Stable CAD MASS III

## Introduction

Coronary bypass surgery performed without the use of cardiopulmonary bypass (off-pump surgery) has been used sporadically since the beginning of the bypass surgery era in 1967, but the use of this strategy increased substantially during the 1990s. The major reason for the increased use of off-pump surgery was the hope that this strategy would decrease perioperative morbidity and possibly mortality by eliminating cardiopulmonary bypass (on-pump surgery). The fear concerning off-pump surgery has been that the difficulty of operating with the heart beating would lead to less-complete and less-effective revascularization at the time of surgery and worse long-term outcomes. These advantages and disadvantages have been examined in several studies that compared the outcomes of patients undergoing off-pump and on-pump surgery [1-4]. Observational trials often have shown bigger differences in short-term complications, usually in favor of off-pump surgery, but analyses of these trials are complicated by patient selection. On the other hand, randomized trials usually have shown small differences in perioperative outcomes, usually slightly in favor of off-pump surgery, mostly including low-risk patients.

Long-term follow-up studies, both randomized and observational, have sometimes noted inferior outcomes after off-pump compared with on-pump surgery, manifested as decreased patency, increased risk of repeat revascularization, or increased mortality. Yet, other studies have shown no or few long-term differences that usually have been attributed to a lack of experience with off-pump surgery [3,4].

The absence of guidelines for the use of one or the other technique has allowed individual decision-making according to the experience of the surgeon [5]. The rationality for off-pump surgery is reduced morbidity, and reduced adverse effects attributed to on-pump surgery, including an inflammatory response caused by the circulation of blood through the cardiopulmonary circuit and the formation of microemboli [6]. In this context, the advantages and disadvantages of both strategies have been considered, and critical appraisal is made of the evidence available. The validity of evidence has been assessed using different criteria, including study design, size of surgical populations, and the quality of statistical analyses [7].

### Objectives

The aim of the MASS III Trial is to compare medical effectiveness, safety, cerebral injury, quality of life, cost-effectiveness of coronary surgery with and without of cardiopulmonary bypass in on-pump and off-pump techniques in patients referred for both strategies. The primary endpoint should be a composite of cardiovascular mortality, cerebrovascular accident, nonfatal myocardial infarction, and refractory angina requiring revascularization.

### Rationale

Coronary artery surgery with and without cardiopulmonary bypass plays an important role in the treatment and management of ischemic heart disease. Both treatment strategies have their own advantages and disadvantages. On-pump strategies provide more complete revascularization and require fewer repeat interventions compared with off-pump surgery. However, the procedure is more invasive and is associated with cardiac as well as noncardiac surgery morbidity [8,9]. Major neurological complications after conventional cardiac surgery have been reported to occur in 3.1% of patients [10]. Additionally, neuropsychological dysfunction is increasingly being recognized as a complication of on-pump surgery. Cognitive deficits can be documented accurately [11] and may occur in up to 38% of patients [12]. The increasing awareness of the cerebral complications following on-pump surgery especially in the elderly [13] has led to a renewed interest in coronary surgery on the beating heart. The Utrecht Octopus method is a technique developed to avoid cardiopulmonary bypass and the complications associated with its use [14].

The role of off-pump surgery, however, is the subject of debate. Opponents emphasize the excellent results of conventional on-pump surgery and express their concerns about the safety, early anastomotic failure, and eventual incomplete revascularization related to off-pump strategies [15].

Moreover, although widely accepted, the suspected deleterious role of cardiopulmonary circulation in the genesis of adverse cerebral outcomes has not been completely proved, simply because an appropriate control group has never been available. A limited number of randomized clinical trials have directly compared coronary surgery with and without cardiopulmonary bypass circulation [16,17].

## Methods

**Figure 1:**
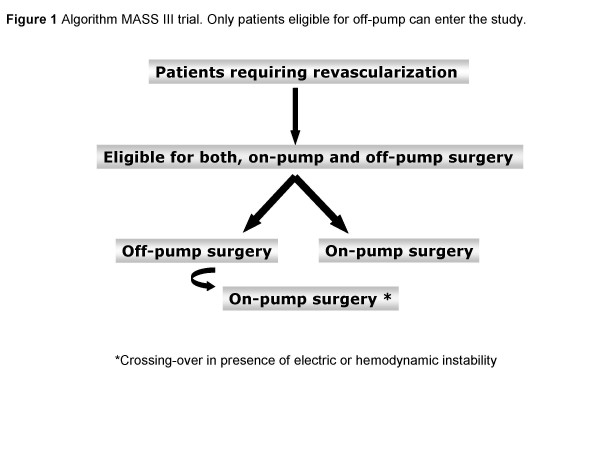
**Algorithm MASS IIItrial.** Only patients eligible for off-pump can enter the study. *Crossing-over in presence of electric or hemodynamic instability.

The MASS III trial is a single-center, prospective, randomized clinical trial, of which the design, timing of investigation and definitions of main outcome events are presented in Figure 1, Table 1 and Table 2, respectively. The MASS III Trial provided external blinded committee. So, all nonfatal clinical events, including MI, Stroke, refractory angina requiring revascularization, will undergo central adjudication by independent Clinical Events Committee (CEC). The role of CEC will be to insure that all primary endpoint are adjudicated uniformly. Furthermore a cardiopulmonary surgeon, a cardiologist and a neurologist formed the clinical event committee and confirm and classify the major adverse cardiac and cerebrovascular events (MACCE), blinded to the treatment. To verify whether important differences in the incidence of MACCE exist between the treatment groups, the Data Monitoring Committee performed an interim analysis after the first patients 100 had entered each arm of study. The three members of this committee are experienced in patient-oriented research, are independent of the study, and may also offer unsolicited recommendations. The sample size calculations are based on the assumptions that the actuarial freedom from cardiac event rate 5 years after on-pump surgery is 95% and that off-pump surgery did not decrease the rate by more than 10%. The α error is set at 0.05, and the β error is set at 0.20. The required sample size is 153 in each group for a total of 306 patients.

**Table 1:** Schedule of Measurements

	S	P	D	1 m	3 m	6 m	12 m	24 m	36 m	48 m	60 m
Angiograms	X		X								X
History/events	X	X	X	X	X	X	X	X	X	X	X
Anginal assessment	X	X	X	X	X	X	X	X	X	X	X
Medications	X	X	X	X	X	X	X	X	X	X	X
Physical examination	X	X	X	X	X	X	X	X	X	X	X
Electrocardiography	X	X	X	X	X	X	X	X	X	X	X
Echocardiography	X										X
Magnetic Resonance Imaging	X		X								
Neuropsychological tests	X		X		X	X					
Routine laboratory	X	X	X	X	X	X	X	X	X	X	X
ECG stress tests	X					X					X
Resource utilization	X	X	X	X		X	X	X	X	X	X
Quality of life	X					X	X	X	X	X	X
Cost-effectiveness		X	X								
Working status	X				X						

Abbreviations: S = preprocedural screening; P = procedure; D = discharge; 1 m = 1 month after intervention; 3 m to 60 m denotes months after intervention.

**Table 2:** Definitions of Main Composite Primary End Points


**Cardiovascular death**	Cardiovascular mortality is included in the composite primary end point. Cardiovascular death includes fatal myocardial infarction, sudden death, untreated heart failure, fatal cerebral infarction, and hemorrhage and procedure-related fatal bleeding.

**Cerebrovascular accident**	Patients with a focal neurological deficit of central origin lasting more than 72 hours, or a focal neurological deficit of central origin lasting more than 24 hours with imaging evidence of cerebral infarction or intracerebral hemorrhage, or a nonfocal encephalopathy lasting more than 24 hours with imaging evidence of cerebral infarction or hemorrhage adequate to account for the clinical state. Retinal arterial ischemia or hemorrhage is also included. To fulfill the definitions of stroke, the deficit must be new, sudden in conset, and not attributable to any more likely alternative cause.

**Myocardial infarction**	Elevation of specific cardiac enzymes within 14 days of a revascularization procedure and presence of new Q waves in at least 2 or more contiguous leads and CK-MB elevation 5 × normal (see Appendix).

**Further revascularization**	The initial revascularization is considered completed when the patient is transferred from the operating room to bed. Refractory angina requiring revascularization was considered an end point.

### Primary Composite End Points

The primary endpoint should be a composite of cardiovascular mortality, cerebrovascular accident, nonfatal myocardial infarction, and refractory angina requiring revascularization across 5 years of follow-up.

It is expected that the need for repeat revascularization after coronary surgery with or without cardiopulmonary bypass will be similar. As mentioned, a controversy discussed in the literature indicates that coronary surgery with or without cardiopulmonary bypass circulation has equal effectiveness in terms of myocardial revascularization. Therefore, repeat interventions can be a major clinical event. Therefore repeat interventions will be considered as early failure of treatment in this study. In addition, the use of cardiopulmonary bypass can be associated with considerable cerebral injury, in particular neuropsychological deficits [11,12]. So, the primary end point in the comparison of surgery with and without cardiopulmonary bypass is also a cerebral event. This is defined as the proportion of patients free of the combined event of fatal and nonfatal cardiovascular accidents and cognitive dysfunction, whichever occurs first, to be determined in-hospital and during the 5 years of follow-up.

### Secondary End Points

The secondary end points in this trial include non-cardiac mortality, presence and severity of angina, quality of life using the SF-36 Questionnaire [18], and cost-effectiveness at discharge and at 5-year follow-up. In this scenario, we will analyze the cost of the initial procedure, hospital length of stay, resource utilization, repeat hospitalization, and repeat revascularization events during the follow-up. Exercise capacity will be assessed at 6-month, 12-month, and end follow-up.

In the MASS III trial, no difference is expected in cardiac outcome; nevertheless, cardiac death, myocardial infarction, and repeat revascularization procedures will be assessed in-hospital and at 1, 6, and 12 months and yearly thereafter. Cognitive function will also be assessed at baseline and at 1, 6, and 12 months. It is expected that most patients will show a decline in this early postoperative period. However, the rapid recovery and short hospital stay of the nonselected patients who undergo coronary surgery in our hospital without extracorporeal circulation suggest that the benefits of this strategy may be especially reflected in a reduction of neuropsychological injury in the early postoperative period.

### Patients

Patients with angiographically documented proximal multivessel coronary stenosis of more than 70% by visual assessment, stable angina, and preserved ventricular function were considered for inclusion in this study. Furthermore, they were eligible if they were referred for isolated coronary bypass surgery for the first time and the off-pump procedure was deemed technically feasible. Patients were enrolled and randomized if surgeons agreed that revascularization could be attained by either strategy. This selection predominantly depends on the precise location of the stenoses, the anticipated capacity of the heart to endure temporary occlusion of the involved coronary arteries, and hemodynamic consequences of local immobilization of the ventricular wall. In these conditions, bypass grafting of posterior coronary arteries may result in a significant drop in left ventricular stroke volume upon presentation of these vessels [19].

All angiograms were reviewed and a surgical plan was documented before randomization. The selection criteria for the MASS III trial are depicted in Table 3.

**Table 3:** Eligibly criteria.


INCLUSION CRITERIA

Male or female age 18 years or older.
Patients with stable angina pectoris and/or documented ischemia due to multivessel disease and preserved ventricular function.
Angiographically confirmed multivessel CAD lesions with ≥70% in at least 2 major epicardial vessels and at least 2 separate coronary artery territories: LAD, LCX, and RCA.
Patients who are eligible for coronary surgery both with and without cardiopulmonary bypass circuit.
Nonsignificant left main stenoses can be included.
Willing to comply with all follow-up study visits.
Signed and received a copy of the informed consent.

EXCLUSION CRITERIA

Age under 18 years
Severe congestive hearth failure NYHA Class III or IV or pulmonary edema.
Prior valve replacement or CABG coronary surgery.
Prior PCI with stent implantation within 6 months.
Prior stroke within 6 months or patients with stroke at more than 6 months with significant residual neurological involvement, as reflected in a Rankin score > 1.
Need for concomitant major surgery, eg, valve replacement, resection ventricular aneurysm, congenital heart disease vascular surgery of the carotid artery, or thoracic-abdominal aorta.
Concomitant medical disorders making clinical follow-up at least 5 years unlikely or impossible, eg, neoplasic, hepatic, or other severe disease.
Q-wave myocardial infarction in the previous 6 weeks.
Hemorrhagic diathesis or hypercoagulability.
Thoracic deformations technically precluding surgery without extracorporeal circulation.
Unable to give informed consent.

### Surgical Technique

#### General

Trial operators were required to perform optimum coronary revascularization in accordance with current best practices. The surgery was performed by physicians experienced in both on-pump and off-pump bypass surgery. Surgical access to the heart was through a standard median sternotomy in all cases. All incisions and closure techniques were the same for groups, limiting variability and maintaining blinding of group assignment for patients, family, and referring cardiologists. A cell saver reservoir (COBE Cardiovascular, Inc. Arvada, CO) was spun down and returned to all patients when the quantity was sufficient.

#### Off-pump strategies

Off-pump surgery used the Octopus stabilizer described in detail elsewhere [14]. In brief, the distal ends of the 2 suction arms of the stabilizer are placed on the beating heart on both sides of the target coronary artery. The proximal parts are fixed to the operating table. Through the application of negative pressure, the target area of the heart is sufficiently immobilized to allow the safe construction of the anastomosis of the graft with the recipient artery.

#### On-pump technique

Conventional coronary artery surgery with cardiopulmonary bypass was accomplished with every effort made to minimize the impact of cardiopulmonary bypass. Patients without diabetes received 1 gram of hydrocortisone sodium succinate (SoluCortef, Pharmacia & Upjohn Co., New York, NY) intravenously before of anesthesia. This procedure will be made only in the on-pump technique. The patients were routinely cooled to 34° for operations with 3 grafts and 32° for 4 grafts or more. Cold-blood cardioplegia was accomplished with anterograde delivery through the aortic root and retrograde delivery through the coronary sinus. A heparinization protocol of 300 U per kilogram for on-pump surgery and half-dose heparin for off-pump surgery was followed. Protamine was used to reverse the effects of heparinization only in the on-pump patients. All anastomoses were sutured by hand. In the off-pump patients, intracoronary shunts were not used routinely; indications for use included poor visibility, ST-segment changes, and homodynamic instability.

### Quality of Life and Cost-effectiveness

Health-related quality of life and treatment costs will be assessed to evaluate the relative cost-effectiveness for the MASS III trial population. Heath-related quality of life and functional status will be assessed using a combination of generic and disease-specific measures selected to cover a broad range of potential health domains that may be affected by coronary artery disease, its treatments, and complications. The SF-36 Questionnaire will be used to assess patients' utilities [18]. Utilities are a global rating of health that reflects a patient's preference for his current health status relative to perfect health and are particularly important outcome measures for cost-effectiveness analysis [20]. Medical care resource utilization and cost data will be collected prospectively for the index hospitalization up to discharge and 5-year follow-up for all patients including all costs associated with the index procedure. For each index revascularization procedure, detailed resource utilization will be collected using a standardized case report form. Follow-up medical resource utilization (including hospitalization, out-patient services, and cardiovascular medications) will be assessed by detailed questionnaires that will be completed during each scheduled patient contact.

### Neurocognitive Evaluation

In evaluation of CABG patients with multivessel disease, neurocognitive status is a crucial outcome variable, along with major adverse cardiac and cerebrovascular event. A subset of subjects will be assessed using the Brain Resource Center [21] computerized neurocognitive battery. The battery is relatively short and easy to administer and score. The computerized battery measures 5 basic functions: memory, mental and psychomotor speed attention, verbal fluency, and cognitive flexibility.

### Statistical Analysis Plan

Patients eligible for the MASS III trial are invited to the outpatient clinic to receive additional information. Candidates for the trial admitted to a referral hospital are visited by one of the trial monitors. After giving consent, patients are randomized. To ensure a reasonable balance, assignment is performed according to a computer-generated list of random permuted blocks that are unknown by the investigators. After randomization, patients are scheduled for the allotted treatment.

The goal of the main analysis is to compare the primary outcome events in this trial. Kaplan Meyer curves will be used for graphic comparison. All values are expressed as mean ± standard deviation or percentage. The continuous variables are compared by the Wilcox rank sum test, whereas the discrete variables are analyzed with Fisher's exact test. The occurrence of outcome events will be compared by means of Cox's proportional hazards model yielding a hazard ratio. The primary data analysis will be based on the intention-to-treat principle. Interim analyses will be carried out annually to evaluate the safety.

For both treatment strategies, the cost of diagnostic procedures, treatment procedures, complications, and short- and long-term differences in effects will be estimated. Marginal costs in monetary terms will be calculated by multiplying unit costs and marginal medical consumption as recorded for each patient.

## Discussion

The MASS III Trial is designed to include patients who need coronary revascularization for relieving angina and better exercise tolerance. Off-pump surgery is a well-established alternative to on-pump surgery in terms of short and mid-term outcomes. However, the benefit of off-pump surgery may depend on the clinical status of patients and the majority of studies were carried out on low-risk surgical patients. Recently, patients with higher risk profile are increasing and in the context of elderly patients with comorbidities, the off-pump surgery might have an important role.

The lack of statement guidelines regarding this issue leaves the decision of using extracorporeal circulation to individual surgeons, and many of them remain unwilling to adopt off-pump surgery technique. This hesitation is still justified, since a more demanding technically procedure is being suggested instead of a successful, well-studied, and reproducible approach. Yet, if the benefits of off-pump surgery became conclusive, this would have several implications for the future of cardiac surgery regarding to quality of patient care as well as cost-effectiveness of the procedure. The MASS III trial is a great opportunity to address all these issues, and it will add relevant information in this field.

### Ethical Considerations

MASS III is conducted in accordance with the principles of the Declaration of Helsinki and with laws and regulations of our country. The Ethics Committee of the Heart Institute of University of Sao Paulo, Brazil approved the study protocol. The attending physician obtained written informed consent from the study participants. The patient is told that he or she will be randomized to surgery with or without extracorporeal circulation.

### Final Considerations

MASS III is designed to include patients who need coronary artery surgery and for whom 2 surgical strategies are feasible. The results of the study may facilitate selection of the most appropriate strategy for individual patients and foster the appropriate use of available resources.

## Competing interests

None of the authors of the MASS III Trial has a financial or any other relation that would pose a conflict of interest.

## Authors' contributions

Each of the authors had substantial contributions either on conception and design or on the drafting of the article and critical revision for this important intellectual content. Specifically, WH is the Principal Investigator for the study described in the manuscript, WH, NHML, BJG, SAO and JAFR participated actively in designing and performing the research. Additionally, SAO, LAD, ACH, and NAS performed all the surgeries procedures. WH and NHML are following all the patients during the follow-up clinical visits. Finally, CCC, FSP, and NHML planed the Ancillaries Studies. All authors participated in drafting and revising the manuscript and all authors read and approved the final manuscript.

## Appendix: Definition of myocardial infarction

**A** – Q-Wave infarction

See ECG-criteria new Q-wave infarction

Enzyme elevation as follows:

CK-MB elevation 5 × upper limit of normal

**B** – Enzymatic/non-Q-wave infarction

Enzyme elevation as follows:

CK-MB elevation 5 × upper limit of normal

**C** – ECG Criteria New Q-Wave myocardial infarction

New QS in 2 associated leads in the absence of left bundle-branch block (LBBB)/Wolf-Parkinson White Syndrome.

New QS in 2 associated leads, defined as ≥ 0.04 seconds broad and/or Q/R ratio ≥ 1/4

Posterior wall infarction: new Broad R-wave (≥ 0.04 seconds) and tall R/wave (R/S ratio▶1 or R-wave ≥ 0.5 mv) in lead V1 and V2 in the absence of right bundle-branch block (RBBB)/right ventricular hypertrophy (RVH)

New permanent LBBB and enzyme elevation

Reversed R-wave progression precordial: decrement R-wave ≥ 0.2 mv in 2 consecutive precordial leads and enzyme elevation.

Any new Q-wave in lead V2 and V3 and enzyme elevation.
